# Trans‐rectovesical pouch urethral‐sparing robotic‐assisted simple prostatectomy: A case series

**DOI:** 10.1002/bco2.389

**Published:** 2024-06-06

**Authors:** Xinnan Chen, Kangkang Zhao, Hao Wang, Chengwei Zhang, Lin Du, Wendi Wang, Tianyi Chen, Haixiang Qin, Xuefeng Qiu, Hongqian Guo, Gutian Zhang

**Affiliations:** ^1^ Department of Urology, Nanjing Drum Tower Hospital, Affiliated Hospital of Medical School Nanjing University Nanjing Jiangsu China; ^2^ Department of Urology, Nanjing Drum Tower Hospital, Affiliated Hospital of Medical School Southeast University Nanjing Jiangsu China; ^3^ Department of Urology The First People's Hospital of Yancheng Yancheng Jiangsu China

**Keywords:** ejaculation function, large volume benign prostatic hyperplasia, trans‐rectovesical pouch, urethral‐sparing robotic‐assisted simple prostatectomy, urethtal‐sparing

## Abstract

**Objective:**

To detail a novel technique of robotic‐assisted simple prostatectomy that makes handling the gland protruding into the bladder neck easier and can preserve the urethra and retain ejaculation function as much as possible.

**Patients and methods:**

This is a prospective case series. Clinical data of 17 male patients who had large volume (>80 mL) benign prostatic hyperplasia (BPH) were enrolled to undergo trans‐rectovesical pouch urethral‐sparing robotic‐assisted simple prostatectomy (usRASP). We adopted the approach through the space between the bladder neck and seminal vesicle to perform a usRASP that can avoid the detrusor skirt and fibrous matrix area of the retropubic prostate. Between the transitional zone and the peripheral zone of the large prostate, the hyperplastic prostatic gland tissue can be enucleated under direct vision while preserving the prostatic urethra and retaining the ejaculatory duct and bladder neck intact. All preoperative, perioperative and postoperative clinical data were collected, and descriptive analysis was performed.

**Results:**

The median intravesical prostatic protrusion was 19.3 mm (8.5–32.2). The median operative time was 100 min (75–140), and the median estimated blood loss was 100 mL (10–500). The median time to catheter removal was 7 days (5–7), with a median postoperative hospital stay of 2 days (2–4). After at least 6‐month follow‐up, the median maximum urine flow rate and postvoid residual volume were 40.1 mL/s (12.7–52.4) and 15 mL (5–23), respectively; the median International Prostate Symptom Score and Quality of Life score were 0 (0–6.3) and 1 (0–3), respectively; and the median total prostate‐specific antigen was 0.84 ng/mL (0.15–1.01). All patients successfully underwent usRASP. Fifty‐eight percent of patients with normal ejaculation function before surgery can still retain normal ejaculation function.

**Conclusion:**

We described a new approach to performing usRASP. This new method remarkably improved the voiding function, maintained antegrade ejaculation and did not increase the post‐operative complications.

## 1 INTRODUCTION

Benign prostatic hyperplasia (BPH) is one of the main causes of lower urinary tract symptoms (LUTS) in men over 50 years of age. In cases where the prostate volume exceeds 80–100 mL, simple prostatectomy (SP) may serve as a viable alternative to transurethral techniques.^1^ Previous studies have shown that minimally invasive methods, such as laparoscopic and robot‐assisted prostatectomy, can offer less blood loss and shorter postoperative hospital stays compared with open SP (OSP).^2^ However, retrograde ejaculation is still a postoperative complication that may influence the patients' quality of life. The rate of retrograde ejaculation after surgery for patients with BPH can reach 50%–70%.^3^ The mechanism underlying the pathogenesis of retrograde ejaculation after surgery may be associated with impaired bladder neck closure and injury in the paracollicular area, prostatic urethra and ejaculatory ducts.^4^

Recent reports of robotic‐assisted SP using the urethra‐sparing technique have shown many advantages, including the preservation of the urethra, bladder neck and ejaculatory ducts, reduced postoperative hematuria and bladder irrigation, having shorter catheterization and postoperative hospital stay, less risk of urethral stricture and, particularly, reduced rates of retrograde ejaculation.^5^ Because of the technical complexity involved in preserving the prostatic urethra, these techniques have not been widely applied. This is because they require extirpating and reconstructing steps, and the disproportionate enlargement of prostate adenoma, along with its lack of support, causes the prostatic urethra to take on an amoeba‐like shape, making it difficult to determine the space between adenoma and urethra.

Therefore, we adopted the approach described by De Concilio et al.^6^ through the space between the bladder neck and seminal vesicle to perform urethral‐sparing robotic‐assisted SP (usRASP). Our preliminary practice results showed that this method can effectively improve the maximum flow rate (Qmax) of patients and reduce the International Prostate Symptom Score (IPSS), and the medial lobe protruding from the bladder can be safely and easily dissected without bladder neck and urethral injury.^7^

Here, we report our surgical details of this innovative technique in a further prospective case series by analysing the clinical data of our patients with large volume (>80 mL) BPH.

## 2 PATIENTS AND METHODS

### 2.1 Study design

This study enrolled consecutively patients with indication for BPH surgery and prostate volume >80 mL between July 2022 and April 2023. These patients underwent usRASP using this technique by one surgeon team at Nanjing Drum Tower Hospital. Before embarking on this attempt, this doctor had more than 1000 cases of robotic surgery for urologic tumours. This study was approved by the ethics committee of Nanjing Drum Towel Hospital (NO. AF/SC‐08/03.0), and the patients provided an informed consent form.

All patients received identical preoperative assessments, including a thorough physical examination, measurement of prostate‐specific antigen (PSA) levels and uroflowmetry, except for patients with an indwelling catheter (*N* = 7). Relevant measurements of prostate volume and postvoid residual (PVR) volume were conducted using suprapubic abdominal ultrasound. In the event of an increased PSA level, individuals were subjected to multiparametric magnetic resonance imaging (mp‐MRI) of the prostate to rule out any indications of potentially malignant prostate cancer (PCA). Follow‐up appointments were scheduled at 1, 3 months and annually following the surgery. The postoperative evaluation included a physical examination, an assessment of PVR volume, uroflowmetry to measure the Qmax and a serum PSA test. Additionally, consenting patients underwent a flexible cystoscopy in an outpatient setting 3 months after the procedure to confirm the structural integrity of the prostatic urethra.

### 2.2 Surgical technique

All the procedures were performed using the Da Vinci Si or Xi Surgical System (Intuitive Surgical, Sunnyvale, CA, USA) in a three‐arm configuration with a transperitoneal approach.

#### 2.2.1 The position of patient, preparation for robot docking via the transperitoneal approach and technique to place trocars

All patients were placed in the supine position and the table in the deep (30°) Trendelenburg position with head and chest flexion upward of 15° after induction of general anaesthesia. Pneumoperitoneum is achieved using a Veress needle inserted in the periumbilical area. Six ports (10 mm port for the optic for SI or XI above the umbilical, three 10 mm ports for robotic instruments and two 12 mm ports for the assistant) were placed in a classical fan configuration. Finally, the robot was docked. The key steps of the operations were shown in a brief video (Videos [Link], [Link], [Link], [Link]).

#### 2.2.2 Preparation for the cleavage plane

**FIGURE 1:**
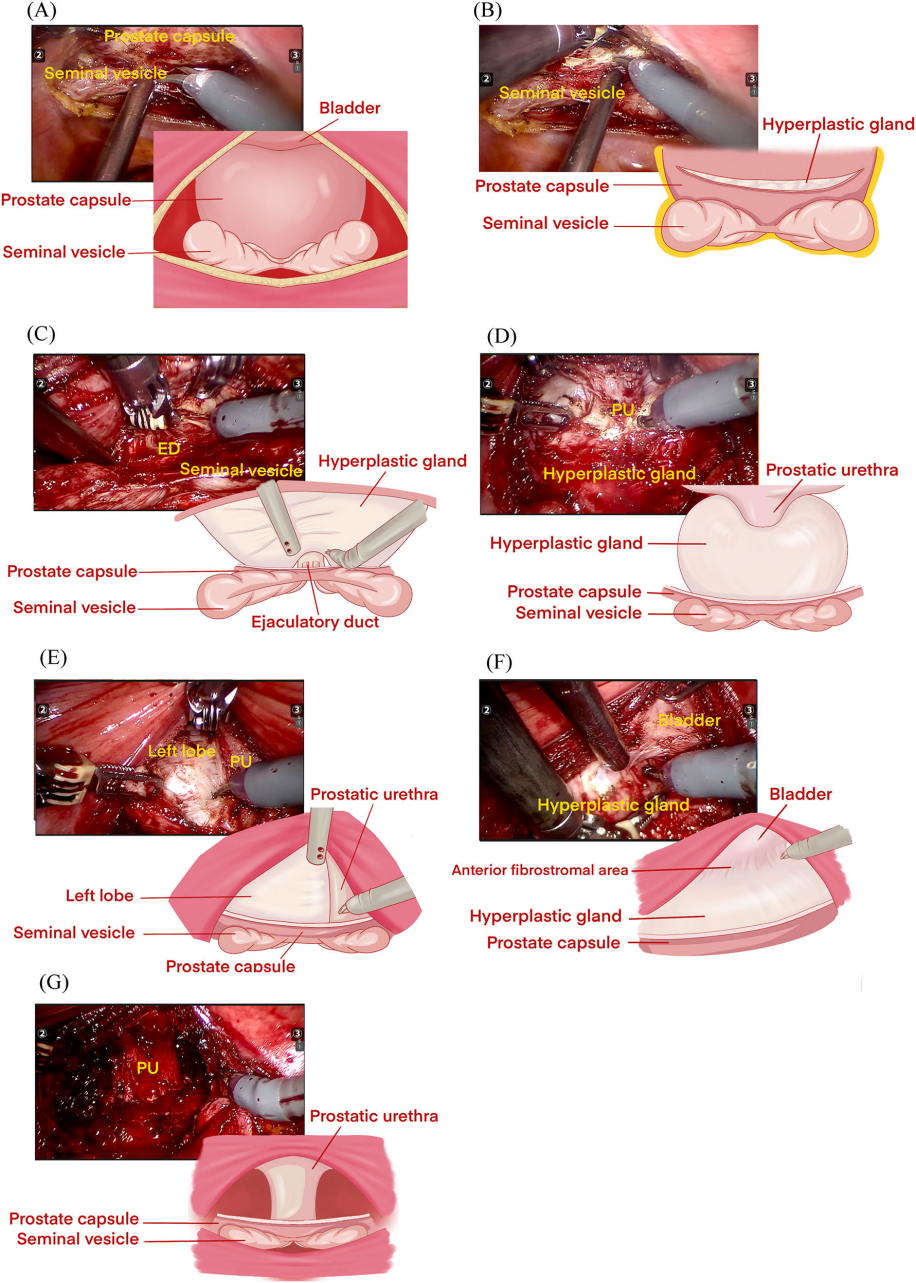
Key steps of trans‐rectovesical pouch urethral‐sparing robotic‐assisted simple prostatectomy. (A) A shallow disc‐shaped incision of the peritoneum of the vesicorectal fossa revealed the prostatic capsule above the seminal vesicle and ejaculatory ducts. (B) A crescent‐shaped transverse incision was made on the prostate capsule. (C) The apex urethra (the area from 3 to 9 o'clock), about 1 cm long, was freed from the apex gland. ED, ejaculatory duct. (D) The bladder neck and the connected prostatic urethra (the area from 1 to 11 o'clock) were freed from the proliferative glands and the middle lobe. PU, prostatic urethra. (E) The left lobe was removed from the prostatic urethra. PU, prostatic urethra. (F) Anterior fibrostromal areas were preserved. (G) The prostatic urethral integrity was tested for any leakage. PU, prostatic urethra.

A shallow disc‐shaped incision of the peritoneum of the vesicorectal fossa revealed the prostatic capsule above the seminal vesicle and ejaculatory ducts (Figure 1A). The transverse incision of the prostate surgical capsule formed a crescent‐shaped incision, exposing the cleavage plane between the proliferative gland and the surgical capsule (Figure 1B).

#### 2.2.3 Dissection of the adenoma

Within the low part of the cleavage plane part of the incision, the hyperplastic glands' posterior, lateral and apex were separated. The apex urethra (the area from 3 to 9 o'clock), about 1 cm long, was freed from the apex gland near the surface. The ejaculatory duct and its urethral junction should be protected (Figure 1C). On the interior part of the incision, both the proliferative glands and the middle lobe were separated and freed from the bladder neck (the area from 1 to 11 o'clock) (Figure 1D). When the volume was extra‐large (more than 180 mL), the incision ends of the prostate surgical capsule could be extended laterally and anteriorly.

The bladder neck and the connected prostatic urethra were elevated with the grasping arm, and the lateral and anterior sides of the hyperplastic gland were carefully dissected along a relatively vascular plane between the surgical capsule of the prostate and the hyperplastic gland, extending to the apex of the prostate. From 1 to 11 o'clock positions, in whole or in parts, removal of the left, right and middle lobe glands was performed, ensuring the preservation of an intact urethra and anterior fibrostromal areas (Figure 1E,F).

#### 2.2.4 Control bleeding, hydrodistension test and close the prostate capsule

After thoroughly removing the hyperplastic glands, bipolar or unipolar coagulation was applied to control bleeding at the apical urethra, bladder neck urethra and prostate fossa to maintain a clear operative field (Figure 1G). When necessary, a posterior midline cystostomy of 3 cm was performed to remove bladder calculi. The prostatic urethral integrity was repaired by suturing, considering the extent of urethral reservation (complete or partial). A size F20 three‐cavity catheter was inserted, and 150 mL of saline solution was instilled through the catheter to assess for any leakage. Finally, the transverse incision in the prostate capsule was closed using a 3‐0 barbed wire with a 16 mm needle.

### 2.3 Data collection

The clinical data included the following patient characteristics: basic information of the patients, prostate volume on MRI, BPH‐related complications (urinary retention/indwelling catheter, bladder stone, hydronephrosis and bladder diverticulum), pre‐ and post‐operative PVR volume, Qmax, IPSS, International Index of Erectile Function (IIEF‐5) score,^8^ total PSA (tPSA) and additional Male Sexual Health Questionnaire to assess Ejaculation Dysfunction (MSHQ‐EjD)^9^ in patients with normal preoperative ejaculatory function.

Perioperative data included operative time, estimated blood and haemoglobin loss, transfusion rate, continuous bladder irrigation, catheterization duration and length of hospitalization. Postoperative complications were also recorded.

### 2.4 Statistical analysis

Continuous variables were summarized as the median and interquartile range. Categorical variables were reported as frequencies (percentages). A non‐parametric test was used to compare the difference between pre‐operation and post‐operation micturition and ejaculation outcomes. A two‐sided value of *p* < 0.05 is considered statistically significant. Data analysis is performed using SPSS version 21.0 software.

## 3 RESULTS

### 3.1 Demographics and preoperative variables

All 17 patients had different degrees of LUTS, and seven of them had relapsed acute urinary retention. The median preoperative prostate volume estimated by MRI images was 127 mL (80–302). The intravesical prostatic protrusion (IPP) of these patients was 19.3 mm (8.5–32.2). Among them, seven patients had indwelling catheters before surgery, and two patients had hydronephrosis. Table 1 displays the demographic and preoperative clinical data.

**TABLE 1:** Baseline demographic and clinical characteristics of 17 patients.

Parameter	Result
Age (year), median (range)	69 (54–84)
BMI (kg/m^2^), median (range)	25 (21–29)
Prostate volume on MRI (mL), median (range)	127 (80–302)
IPP (mm), median (range)	19.3 (8.5–32.2)
BPH‐related complications, *n* (%)	
Urinary retention/indwelling catheter	7 (41.2)
Bladder stone	0 (0)
Hydronephrosis	2 (11.8)
Bladder diverticulum	0 (0)

Abbreviations: BMI, body mass index; BPH, benign prostatic hyperplasia; IPP, intravesical prostatic protrusion; MRI, magnetic resonance imaging.

### 3.2 Perioperative findings

The intraoperative findings unveiled the subsequent:

- Five cases (29.41%) underwent a complete adenomectomy while preserving the urethra.
- Urethral‐sparing adenomectomy was performed with minimal urethral injury, necessitating the use of specialized 4/0 monofilament sutures for precise stitching in 10 cases (58.82%).
- Failed urethral‐sparing adenomectomy, converted to continuous vesicourethral anastomosis, was observed in two cases (11.76%); in two of these cases, it took place during the removal of the lobe located in front of the apical urethral.

The procedures were not converted to open or pure laparoscopic SP.

The median tissue enucleated was estimated to be 79 mL (35–198) based on the weight of the removed tissue. As shown in Table 2, the median operative time was 100 min (75–140), the median blood loss was 100 mL (10–500), the median haemoglobin decreased by 1.9 g/dL (0–3.8) on the first day after surgery, the median length of postoperative hospitalization was 2 days (2–4) and the median time to catheter removal was 7 days (5–7). Two instances (11.7%) had transfusion therapy belonging to the Grade 2 Clavien–Dindo complication, and no other complications occurred in the other patients.

**TABLE 2:** Perioperative and pathological outcomes.

Parameter	Result
Operative time (min), median (range)	100 (75–140)
Estimated blood loss (mL), median (range)	100 (10–500)
Estimated haemoglobin loss (g/dL), median (range)	1.9 (0–3.8)
Transfusion, *n* (%)	2 (11.7)
Continuous bladder irrigation, *n* (%)	0 (0)
Time to catheter removal (d), median (range)	7 (5–7)
Length of stay (postoperative) (d), median (range)	2 (2–4)
Enucleated prostate tissue (mL), median (range)	79 (35–198)
Conversion to standard technique, *n* (%)	0
Clavien–Dindo complications, *n* (%)	
Grade 1	0
Grade 2	2 (11.8)
Grade 3a	0
Grade 3b	0
Incidental prostate cancer, *n* (%)	0

### 3.3 Postoperation micturition and ejaculation outcomes

**FIGURE 2:**
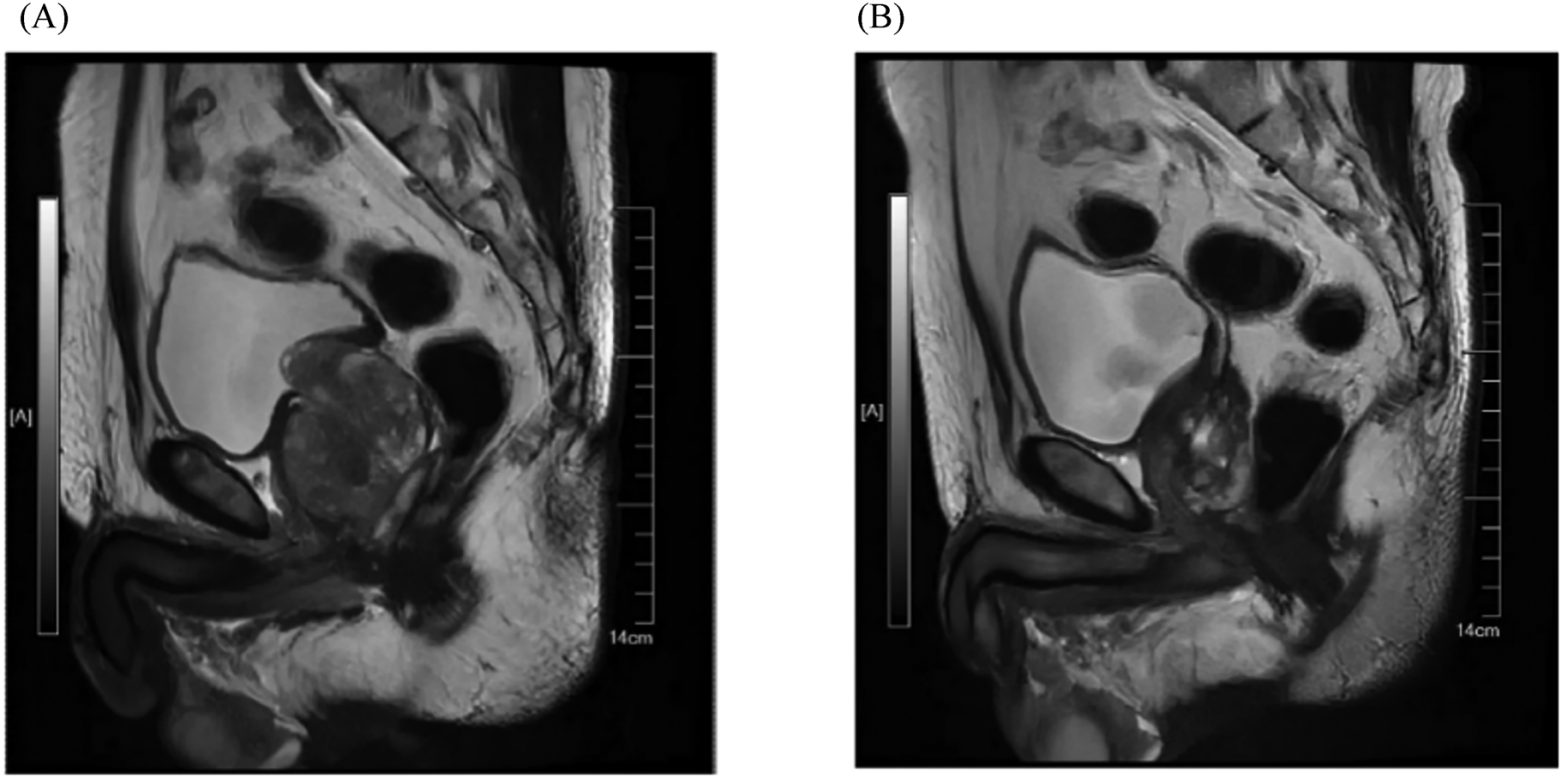
The magnetic resonance imaging (MRI) in sagittal plane of the prostate for the same patient before the operation (A) and 1 year after operation (B).

Functional outcomes are shown in Table 3; the median follow‐up was 9 months (6–12), and all the patients had significantly improved urination symptoms. The median preoperative values of the IPSS and quality of life (QoL) questionnaires were 29 (7–35) and 6 (3–6), respectively, and the median postoperative values were 7 (1–10) and 1 (0–3), respectively. Median preoperative Qmax was 0 mL/s (0–6.3) and postoperative measurement was 40.1 mL/s (12.7–52.4). The median preoperative PVR volume was 160 mL (66–284), and the median postoperative measurement was 15 mL (5–23). The median preoperative and postoperative values of tPSA were 7.79 (1.46–29.6) and 0.84 ng/mL (0.15–1.01). Fifty‐eight percent of patients (7/12) with normal ejaculation function before surgery can still retain ejaculation function; there was no significant difference in the questionnaire results of patients with preserved ejaculatory function. Figure 2 indicates the MRI of the prostate for the same patient before the operation and 1 year after the operation. A video of the postoperative cystourethroscopy of one of the patients is submitted in Video S5.

**TABLE 3:** Functional outcomes2.

	Preoperative, median (range)	Postoperative, median (range)	*p* value
Qmax (mL/s)	0 (0–6.3)	40.1 (12.7–52.4)*	0.003
PVR volume (mL)	160 (66–284)	15 (5–23)*	0.025
IPSS	29 (7–35)	7 (1–10)*	0.000
QoL	6 (3–6)	1 (0–3)*	0.000
IIEF	19 (5–23)	17 (4–23)	0.569
MSHQ‐EjD	13 (7–14)	11.5 (4–14)	0.212
tPSA (ng/mL)	7.79 (1.46–29.6)	0.84 (0.15–1.01)*	0.000

Abbreviations: IIEF, International Index of Erectile Function; IPSS, International Prostate Symptom Score; MSHQ‐EjD, Male Sexual Health Questionnaire to assess Ejaculation Dysfunction; PVR, postvoid residual; Qmax, maximum flow rate; QoL, quality of life; tPSA, total prostate‐specific antigen.

* *p* < 0.05 versus before operation.

## 4 DISCUSSION

We described an innovative method of usRASP. Our follow‐up findings that the novel technique significantly improved the urinary function outcomes after the operation indicate that trans‐rectovesical pouch usRASP may be a safe and effective treatment for BPH patients with large volumes. The median IPP of these patients was 19.3 mm (8.5–32.2), and all patients were successfully treated, suggesting that the technique may make handling the gland that protrudes into the bladder easier. This technique may be a good solution to postoperative retrograde ejaculation based on the fact that 58% of patients with normal ejaculation before surgery can still retain ejaculation function.

For patients with large volume BPH who require surgical removal of the obstruction, OSP and holmium laser enucleation of the prostate (HoLEP) are effective surgical options traditionally.^10^ Madigan and colleagues first reported Madigan SP through an open approach with the urethra sparing technique.^11^ The complete structure of the urethra can be preserved during the operation, and antegrade ejaculation can be maintained after the operation without affecting orgasm and sexual satisfaction. In 2011, through a laparoscopic approach, Madigan SP showed the same benefit and less invasion compared with operation through an open approach.^12^ However, the Madigan SP is not widely used because of the complexity of the technique and the difficulty in performing under ordinary laparoscopy, especially in the case of middle lobe prostatic protrusion. In the current era of robot‐assisted surgical procedures that enhance visualization, dissection and suturing capabilities, there has been a proposal to increase the utilization of techniques aimed at preserving the urethra. However, Wang et al.,^13^ Simone et al.^14^ and Porpiglia F. et al.^5^ have recently proposed a new method for robot‐assisted prostatectomy to preserve the urethra. Nevertheless, these studies had certain limitations, including a lack of consensus in outcome standardization, the requirement to remove the median prostatic lobe by opening the bladder neck and inadequate reporting of specific surgical details.

In this study, we utilized the trans‐rectovesical pouch to perform usRASP for high‐volume benign prostatic hyperplasia. Originally used in oncological surgery, this novel surgical technique enables a more effective adenomectomy with bladder neck sparing, leading to improved functional outcomes. We have found that by making a transverse incision on the posterior and upper prostate capsule (the space before the seminal vesicle), the protruding central zone, which is only present in patients with large volume BPH, can be directly exposed to the surgical field without having to remove the median prostatic lobe by a cystotomy. Our findings demonstrate the effectiveness of this approach, which is comparable to that of other researchers. The technique significantly improved voiding function, maintained antegrade ejaculation and did not result in increased postoperative complications.^15^, ^16^, ^17^ In two cases, failed urethral‐sparing adenomectomy was converted to continuous vesicourethral anastomosis because of the difficulty in removing the lobe located in front of the apical urethra and urethral adhesion. Their postoperative recovery was smooth and did not require bladder irrigation.

No control group, single‐centre trial, relatively short follow‐up period and small sample size are the major limitations of this study. This study is a preliminary exploratory case series. Nevertheless, this study has presented a novel technique of usRASP that can handle the gland protruding into the bladder neck and can preserve the urethra and retain ejaculation function as much as possible.

## 5 CONCLUSION

The trans‐rectovesical pouch usRASP is a safe and effective treatment for BPH patients with large prostates, especially for patients with a gland that protrudes into the bladder. This technique can completely or partially preserve the prostatic urethra, remove the obstruction and preserve the ejaculation function simultaneously.

## AUTHOR CONTRIBUTIONS

**Xinnan Chen**: Data curation; formal analysis; writing—original draft; writing—review and editing; visualization. **Kangkang Zhao**: Data curation; writing—review and editing; visualization. **Hao Wang**: Data curation. **Chengwei Zhang**: Data curation; writing—review and editing; resources. **Lin Du**: Writing—review and editing. **Wendi Wang**: Data curation. **Tianyi Chen**: Data curation. **Haixiang Qin**: Resources. **Xuefeng Qiu**: Resources. **Hongqian Guo**: Resources; visualization. **Gutian Zhang**: Conceptualization; methodology; validation; writing—review and editing.

## CONFLICT OF INTEREST STATEMENT

None of the contributing authors have any conflicts of interest, including specific financial interests and relationships and affiliations relevant to the subject matter or materials discussed in the manuscript.

## Supporting information


**Video S1.** Video S1

**Video S2.** Video S2

**Video S3.** Video S3

**Video S4.** Video S4

**Video S5.** Video S5

## Data Availability

The corresponding author had full access to all the data in the study and takes responsibility for the integrity of the data and the accuracy of the data analysis.
